# Synthesis, Structure, and UV–Vis Characterization of Antimony(III) Phthalocyanine: [(SbPc)_2_(Sb_2_I_8_)(SbBr_3_)]_2_

**DOI:** 10.3390/molecules27061839

**Published:** 2022-03-11

**Authors:** Ryszard Kubiak, Jan Janczak

**Affiliations:** Institute of Low Temperature and Structure Research, Polish Academy of Sciences, Okólna 2, 50-422 Wrocław, Poland; r.kubiak@intibs.pl

**Keywords:** antimony(III) phthalocyanine, crystal structure, Hirshfeld surface, oxidation, UV–Vis spectroscopy

## Abstract

A new antimony(III)–phthalocyanine complex with the formula of [(SbPc)_2_(Sb_2_I_8_)(SbBr_3_)]_2_ has been obtained in the reaction of pure antimony powder with phthalonitrile under the oxidation conditions by iodine monobromide vapors. The complex crystallizes in the centrosymmetric space group of the triclinic system. Both independent (SbPc)^+^ units exhibit non-planar conformation, since the Sb(III) is larger than the equilibrium cavity size of the ring and cannot be accommodated without its expansion; thus, the metal protrudes out of the cavity, forming a saucer shape. The centrosymmetric anionic unit of the crystal consists of two (Sb_2_I_8_)^2−^ interacted anionic units forming (Sb_4_I_16_)^4−^ anionic complex that interacts with two SbBr_3_ molecules to form [Sb_6_I_16_Br_6_]^4−^ anionic aggregate. Each [Sb_6_I_16_Br_6_]^4−^ anionic aggregate is surrounded by four (SbPc)^+^ cations forming a supramolecular centrosymmetric (SbPc)_4_[Sb_6_I_16_Br_6_] complex. Translationally related (SbPc)_4_[Sb_6_I_16_Br_6_] molecules form a stacking structure along the [100] and [011] directions with N_4_–N_4_ distances of 3.55 and 3.53 Å, respectively, between the back-to-back-oriented saucer-shaped (SbPc)^+^ units. The interaction between the building units of the crystal was analyzed using the Hirshfeld surface and the analysis of the 2D fingerprint plots. The UV–Vis absorption spectra of crystal **1** were taken in CH_2_Cl_2_ and toluene solutions in the concentration range from 10^−5^ to 10^−6^ mol/L. No significant changes related to aggregation in solutions were observed. The Q-band in toluene solution is red shifted by ~15 nm in comparison to that in CH_2_Cl_2_ solution. Oxidation of (SbPc)_4_[Sb_6_I_16_Br_6_] yields Sb^V^Pc derivative. Both Sb^III^ and Sb^V^ phthalocyanine derivatives absorb near infrared light (600–900 nm), which should be intriguing from the point of view of potential use as photosensitizers for PDT and as an infrared cut filter for plasma display and silicon photodiodes.

## 1. Introduction

**Scheme 1:**
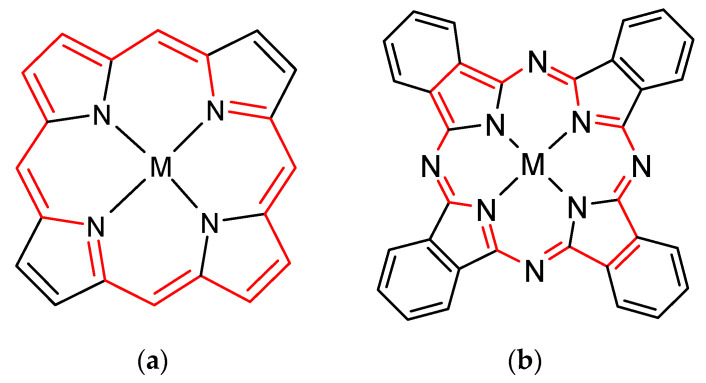
Metalloporphyrins (**a**) and metallophthalocyanines (**b**) showing the 18π-electron tetrapyrrole macrocycle (in red). For metal-free porphyrin or metal-free phthalocyanine, M is two hydrogens.

Phthalocyanine and its metal complexes are still very intensively studied due to their various technological applications. The initial interest in them resulted from their deep and intense color; hence, they were used as pigments in the plastic industry and as dyes in the textile industry [1,2,3,4,5,6,7,8,9]. The dyeing properties of metallophthalocyanines result from their structure and very strong characteristic optical absorption in the visible region, the so-called Q band, which is characteristic for a macrocyclic tetrapyrrole ring with an 18π-electron-conjugated tetrapyrrole macrocycles (Scheme 1) [10]. The molar extinction coefficient of the Q-band of metal-free porphyrin, is 1.71 × 10^5^ dm^3^·mol^−1^·cm^−1^ at 407 nm, and for metal-free phthalocyanine it is 2.766 × 10^5^ dm^3^·mol^−1^·cm^−1^ in the visible spectral region (669 nm), and they may vary significantly depending on the solvents [11]. For metalloporphyrins and metallophthalocyanines, the molar extinction coefficient varies depending on the central metal as well as on the solvent in the wide range, for example, for the zinc porphyrin and zinc phthalocyanine the molar extinction coefficient is 5.74 × 10^5^ dm^3^·mol^−1^·cm^−1^ at 422.8 nm and 2.818 × 10^5^ dm^3^·mol^−1^·cm^−1^ at 674 nm, respectively [12,13]. The molar extinction coefficients of several metal(II) phthalocyanines in various solvents derived from the intensities of the Q-band were recently published [14].

**Scheme 2:**
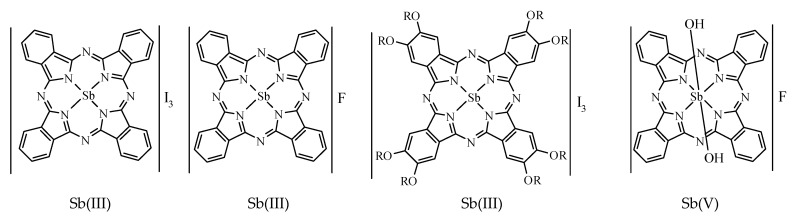
Examples of some Sb(III) and Sb(V) phthalocyanines (-OR = C_6_H_5_O- or *tert*-bythylO-).

For antimony(III)–phthalocyanine derivatives (Scheme 2) in ethanol solution, the molar extinction coefficient is 1.059 × 10^5^ and 1.717 × 10^5^ dm^3^·mol^−1^·cm^−1^ at ~730 nm for unsubstituted Sb(III) phthalocyanine and 1.06 × 10^5^ dm^3^·mol^−1^·cm^−1^ at ~740 nm for octa-substituted Sb(III) phthalocyanine, whereas for Sb(V) phthalocyanine, the molar extinction coefficient is 1.908 × 10^5^ dm^3^·mol^−1^·cm^−1^ at 699 nm [15,16].

Further interest of metallophthalocyanines results from their interesting properties, such as electronic, photonic, and photovoltaic that have already found industrial applications in many instruments or chemical processes, including catalysis, gas sensors, solar cells, charge-generating materials, optical data storage, infrared cut-off filters, active matrix displays, light organic-emitting diodes (OLED), organic photovoltaics (OPVs), etc. [17,18,19,20,21,22,23,24,25,26,27]. Strong and intense absorption of light by metal phthalocyanines in the so-called therapeutic window, namely in the near-infrared (NIR) region of visible light (600–900 nm), proved to be promising for photodynamic cancer therapy (PDT) in which they can be used as photosensitizers [28,29,30,31,32,33,34,35].

The physical and chemical properties of metallophthalocyanines are varied by the structure of the macrocyclic ligand with the extended π-electron delocalization system as well as by the central metal. Planar metallophthalocyanines with 18π electrons conjugate form a stacking structure in the solid state stabilized by π–π interactions, and this limits their solubility in the most commonly used organic solvents and undergo molecular aggregation [36]. Aggregation phenomena are common in chemistry of phthalocyanines and are known to give rise to significant changes in their physical properties [37,38,39] and may arouse interest from the point of view of supramolecular chemistry [40]. Metallophthalocyanines containing heavy atoms with too large radii to fit into the phthalocyanine core are nonplanar and lead to the reduction of π–π interactions and improve the intersystem crossing [41], resulting in the improvement in photophysical and photochemical properties as well as improve their solubility due to the reduction of molecular aggregation, resulting from changes in the electronic structure of the molecule as well as due to the dipole moment of the non-planar molecule [42].

A large number of metallophthalocyanine derivatives have so far been studied; however, the metallophthalocyanines with group 15 elements, unlike porphyrins analogues [43,44,45,46,47], were ignored for a long time until the first derivatives of bismuth and antimony were identified [48,49,50,51], and their structural characterizations are still less explored [52,53,54,55,56,57,58,59,60,61,62,63,64]. The variable valency of these metals depending on the reaction conditions (III or V) can lead to the formation of various MPc derivatives. SbPc derivatives can be promising candidates for new drugs because they are stable, powerful electron donors/acceptors [65,66], and some derivatives undergo facile Sb^III^/Sb^V^ conversion under mild conditions [67,68]. Their use as drugs requires further research on their biocompatibility and effectiveness as well as their toxicity.

In this work, we report synthesis and X-ray structural characterization of a novel antimony(III) phthalocyanine, [(Sb^III^Pc)_2_(Sb_2_I_8_)(SbBr_3_)], obtained under an iodine monobromide vapor atmosphere. The Sb^III^ cation with a radius of about 0.90 Å [69] is larger than the size of the ring cavity and cannot be accommodated without ring expansion; therefore, the formation of non-planar derivatives of Sb^III^Pc can be expected. The antimony(III)–phthalocyanine complexes can be oxidized to Sb^V^Pc derivatives. In addition, the obtained antimony(III) phthalocyanine and its oxidized Sb^V^Pc derivative, as described below, exhibits absorption of a wide range of visible light. Therefore, this should be of interest to those who work not only with dyes and pigments but also with solar cells and charge-generating material. It should be noted that antimony-phthalocyanine derivatives due to strong absorption of light in the therapeutic window may be attractive from the viewpoint of their potential application as a photosensitizer for PDT [70,71,72,73,74].

## 2. Experimental Section

### 2.1. General Procedure

Phthalonitrile (98%), iodine monobromide (98%), and antimony (99.999%) were obtained from Merck (Darmstadt, Germany) and used as received. The composition of the obtained crystals was checked with energy dispersive spectroscopy (EDS) (FEI company, Thermo Fisher Scientific, Waltham, MA, USA). EDS spectra were acquired and analyzed using an EDAX Pegasus XM4 spectrometer with an SDD Apollo 4D detector mounted on a FEI Nova NanoSEM 230 microscope. In addition, the elemental analysis was also carried out with a PerkinElmer 240 elemental analyzer (Waltham, MA, USA). The Fourier transform infrared spectra were recorded between 4000 and 450 cm^−1^ on a Bruker IFS 113 V FTIR (Bruker Optik GmbH, Leipzig, Germany) in nujol mull (Figure S1, in Supplementary Materials). Measurements of the UV–Vis spectra were carried out at room temperature using an Agilent UV–Vis/NIR Cary 5000 spectrometer (Agilent Technologies, Inc., Santa Clara, CA, USA). The UV–Vis spectra were recorded in CH_2_Cl_2_ and toluene solutions with concentrations ranging from 1 × 10^−5^ to 1 × 10^−6^ mol/L.

### 2.2. Synthesis [(Sb^III^Pc)_2_(Sb_2_I_8_)(SbBr_3_)]_2_

The crystals of the [(Sb^III^Pc)_2_(Sb_2_I_8_)(SbBr_3_)]_2_ (**1**) were isolated as a product of the reaction of Sb (0.1218 g, 1.0 mmol) with phthalonitrile (0.51252 g, 4 mmol) under iodine monobromide vapor according to a slightly modified method described elsewhere [75]. The powder Sb and phthalonitrile (in a molar proportion of 1:4) were mixed with iodine monobromide (2.068 g, 10.0 mmol) and pressed into the pellets. The pellets were inserted into an evacuated glass ampoule and sealed. The ampoule was heated at 240 °C for one day, and next, it was cooled to room temperature. After such a process, good quality single crystals of [(Sb^III^Pc)_2_(Sb_2_I_8_)(SbBr_3_)] suitable for the X-ray analysis were formed. Anal. Found: C, 26.51; Br, 8.26; I, 35.21; N, 7.72, and Sb, 21.20%. Calc. for C_64_H_32_Br_3_I_8_N_16_Sb_5_: C, 26.61; Br, 8.30; I, 35.14; N, 7.76; Sb, 21.07; and H, 1.12%.

### 2.3. X-ray Crystallography

X-ray intensity data collection for the [(Sb^III^Pc)_2_(Sb_2_I_8_)(SbBr_3_)]—(**1**) crystal was collected using graphite monochromatic MoKα radiation on a four-circle *κ* geometry Xcalibur diffractometer with Sapphire2 area CCD detector. Data collections were made using the CrysAlis CCD program [76]. Integration, scaling of the reflections, correction for Lorenz and polarization effects, and absorption corrections were performed using the CrysAlis Red program [76]. The structures were solved by the direct methods using SHELXT-2014/7 [77] and refined using the SHELXL-2018/3 program [78]. The hydrogen atoms joined to aromatic carbon atoms were introduced in their geometrical positions and treated as rigid. The final difference Fourier maps showed no peaks of chemical significance. Details of the data collection parameters, crystallographic data, and final agreement parameters are collected in Table 1. Visualizations of the structures were made with the Diamond 3.0 [79].

### 2.4. Hirshfeld Surface Analysis

Hirshfeld surface analysis and 2D fingerprint plots as well as percentage contributions for various intermolecular contacts in the investigated crystals, were calculated using the Crystal Explorer Ver. 3.1 program package [80].

### 2.5. Theoretical Calculations

The optimized geometry of the (SbPc)^+^ unit was performed based on the experimental X-ray geometry using the DFT and PBEPBE functional and LAN2MB basis sets using the Gaussian 2016 program package [81].

## 3. Results and Discussion

### 3.1. Synthesis and Initial Characterization

**Scheme 3:**
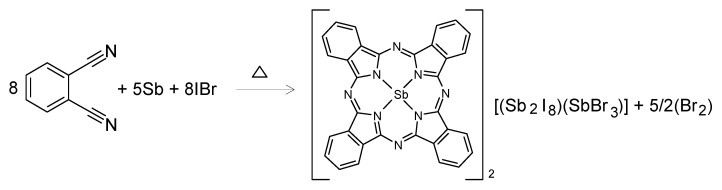
Synthesis of antimony phthalocyanine under oxidation condition of iodine monobromide vapor.

The preparation method leads directly to good-quality [(Sb^III^Pc)_2_(Sb_2_I_8_)(SbBr_3_)]—(**1**) single crystals. During prolonged heating, the liquid phthalonitrile undergoes catalytic tetramerization forming the phthalocyanine units while accepting the two electrons from the antimony present in the reaction forming (Sb^III^Pc)^+^ cationic units (Scheme 3). Simultaneously, the iodine monobromide accepted the third electron as well as oxidized the excess of antimony present in the reaction forming the centrosymmetric {[(Sb_2_I_8_)(SbBr_3_)]_2_}^4−^ anionic complex. These oppositely charged units interact with each other, leading to nucleation and, over time, to crystallization and growth of good-quality single crystals suitable for X-ray diffraction.

It should be noted that the heating time should not be extended too long, as it leads to partial decomposition of the phthalocyanine complex formed and to the formation of the phthalonitrile trimer [82]. Elemental analysis of the obtained crystals gave a satisfactory result, which is close to the calculated one. The sharp peak for the C≡N stretching vibrations in the IR spectrum of phthalonitrile at ~2200 cm^−1^ disappeared after tetramerization into phthalocyanine moiety [83,84]. The IR spectrum of (SbPc)(I_3_) ½(I_2_) (Figure S1 in Supplementary Materials) showed vibration peaks between 700 cm^−1^ and 1500 cm^−1^, which can be attributed to the skeletal vibration of the phthalocyanine macrocycle [85]. The obtained [(Sb^III^Pc)_2_(Sb_2_I_8_)(SbBr_3_)] crystals have limited solubility in methanol and ethanol and dissolve much better in pyridine, DMF, DMSO, toluene, benzene, and other aromatic solvents. Contact with concentrated inorganic acids, such as H_2_SO_4_ or HNO_3,_ or their mixture or mixture with concentrated HCl leads to demetallation, resulting in the formation of metal-free phthalocyanine in the α-form [86] and appropriate salt.

### 3.2. Structural Characterization

**Figure 1:**
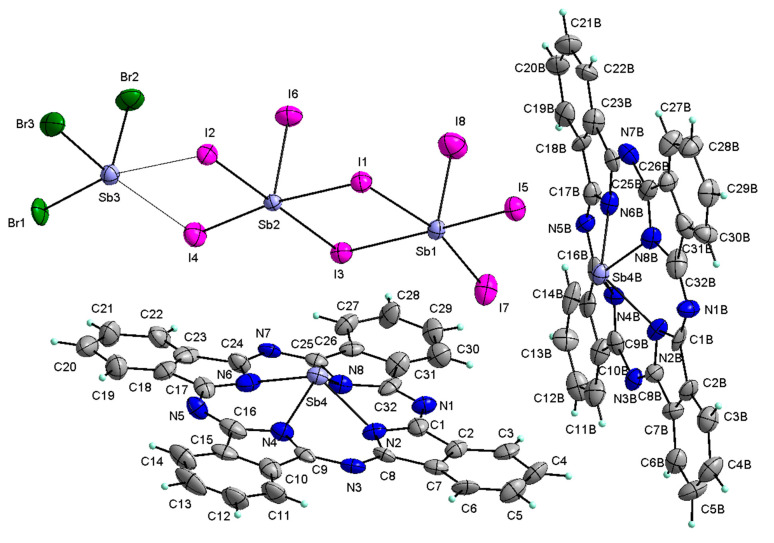
Asymmetric unit of **1** showing two independent (SbPc)^+^ units and (Sb_3_I_8_Br_3_)^2−^.

The obtained antimony-phthalocyanine complex crystallizes in the centrosymmetric space group of the triclinic system. Refinement of the structure of the title compound (**1**) shows that both ionic parts of the crystal building blocks, i.e., in (SbPc)^+^ as well as in the [(Sb_2_I_8_)(SbBr_3_)]^2−^, contain the antimony in the same oxidation state (Sb^3+^). The molecular structure of the asymmetric unit is illustrated in Figure 1.

Both independent (SbPc)^+^ units exhibit similar non-planar conformation, since the Sb^3+^ (ionic radius of about 0.90 Å [69]) is larger than the equilibrium cavity size of the ring and cannot be accommodated without ring expansion; thus, the metal protrudes out of the Pc-cavity, forming a domed shape. Each of the positively charged atoms, Sb4 and Sb4B, of the cations (SbPc)^+^ is bound to four isoindole N atoms of Pc macrocycles with distances in the range from 2.146(8) to 2.206(8) Å (Table 2) and lies outside the plane defined by these N atoms, i.e., 0.992(1) and 0.994(1) Å for Sb4 and Sb4B, respectively. The optimized geometry of the (SbPc)^+^ unit performed by DFT calculations also show its non-planar conformation with the Sb atom displaced by 0.886 Å out of N_4_-isoindole plane (See Table S1 in Supplementary Materials). The greater displacement of Sb(III) from the N_4_-plane in the crystal is caused by the interaction of the positively polarized Sb^3+^ center of (SbPc)^+^ with the (Sb_6_I_16_Br_6_)^4−^ counterion.

**Figure 2:**
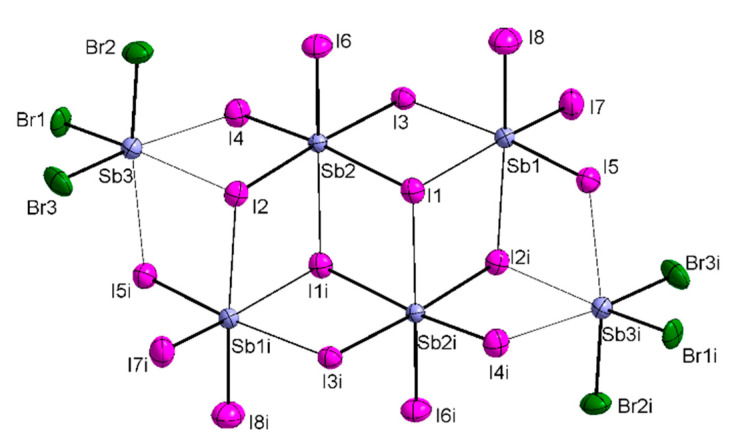
View of the anionic unit of **1**.

The anion of the complex consists of four distorted SbI_6_ octahedra lined together by μ_2_ and μ_3_ bridging I atoms into a (Sb_4_I_16_)^4−^ anion that weakly interacts with two SbBr_3_ molecules forming the centrosymmetric (Sb_6_I_16_Br_6_)^4−^ counterion complex (Figure 2). Generally, the Sb–I bond lengths fall into two groups, namely shorter Sb–I bonds with terminal I atoms and longer Sb–I bonds involving the bridging I atoms (Table 2). However, in the (Sb_6_I_16_Br_6_)^4−^ anion, two different bridging I atoms exist. Atoms I1 and I2 bridge three Sb atoms, while atoms I3, I4, and I5 bridge only two Sb atoms. The Sb–Br bonds with distances ranging from 2.5659(14) to 2.6888(12) Å are shorter, as expected, than the Sb–I bonds (Table 2) and correlate with their covalent or ionic radii of I and Br [87,88]. Looking in more detail at the differences between the Sb–I and Sb–Br bond lengths and at the coordination geometry around atoms Sb1, Sb2, and Sb3, it is clear the Sb1 and Sb2 atoms are coordinated to the six I atoms through forces of different strength, whereas Sb3 coordinates to three Br and the three I atoms. Atom Sb1 links two I atoms with relatively short Sb–I bonds, two I atoms with intermediate Sb–I values, and two I atoms with relatively long Sb–I bonds, the longest being the Sb1*^i^*–I1 bond 3.4281(12) Å (Table 2).

The Sb2 atom in the coordination environment has one terminal I atom with the short Sb–I bond (2.7374(11) Å), two μ_2_-bridged, and three μ_3_-bridged I atoms with intermediate and longest Sb–I bonds (Table 2). The Sb3 links three Br atoms, all Br are terminal, with relatively short Sb–Br bond lengths, and the two μ_2_-bridged and one μ_3_-bridged I atoms with relatively long Sb–I bonds above 3.3 Å. Thus, the [Sb_6_I_16_Br_6_]^4−^ ion can be regarded as being composed of three symmetrically equivalent pairs of units, viz. [SbI_5_]^2−^, SbI_3,_ and SbBr_3_ units. However, the mutual orientation of the [SbI_5_]^2−^, SbI_3,_ and SbBr_3_ units related by an inversion center leads to the formation of an [Sb_6_I_16_Br_6_]^4−^ counter-ion in which all Sb atoms have distorted octahedral coordination environments (Figure 2).

A similar pattern concerning the Sb–I bond lengths is also observed in the [Sb_4_I_16_]^4−^ [59], [Sb_4_I_14_]^2−^ [62], and [Sb_6_I_22_]^4−^ [63] counter-ions of antimony(III)–phthalocyanine complexes. In the first complex, the [Sb_4_I_16_]^4−^ ion consists of four distorted SbI_6_ octahedra, in the second, the [Sb_4_I_14_]^2−^ ion consists of two pairs of deformed SbI_6_ octahedra and distorted square pyramidal SbI_5_ polyhedra, while in the third, the [Sb_6_I_22_]^4−^ ion consists of six distorted SbI_6_ octahedra similar as in the present compound in which the Br atoms have been replaced by the I atoms. A similar metal-polyheterohalide anion can be found in the bismuth(III)–phthalocyanine derivative whose [Bi_6_Cl_11_I_11_]^4−^ anion consists of edge-shared distorted octahedra BiI_3_Cl_3_ [64].

**Figure 3:**
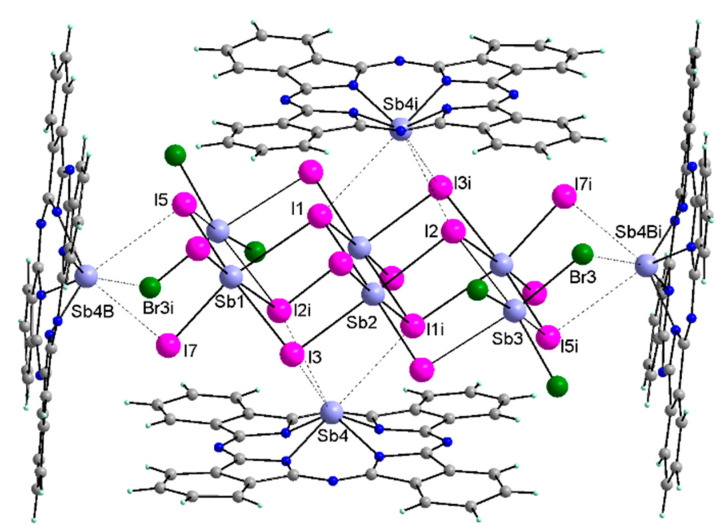
View of the anionic [Sb_6_I_16_Br_6_]^4−^ complex surrounded by four (SbPc)^+^ in the crystal of **1**.

In the crystal, there seems to be significant ionic attraction between the (SbPc)^+^ cations and [Sb_6_I_16_Br_6_]^4−^ complex counterions. The influence of the ionic attraction between the oppositely charged (SbPc)^+^ and [Sb_6_I_16_Br_6_]^4−^ ions is clearly manifested in the Sb–N(isoindole) coordination, leading to the molecular symmetry of the (SbPc)^+^ unit being close to *C_s_*, rather than the *D_4v_* symmetry that characterizes the conformation in solution. The basic packing unit includes two pairs of (SbPc)^+^ macrocycles associated by an inversion centre and an anionic [Sb_6_I_16_Br_6_]^4−^ complex; thus, each anion is surrounded by four (SbPc)^+^ cations (Figure 3).

**Figure 4:**
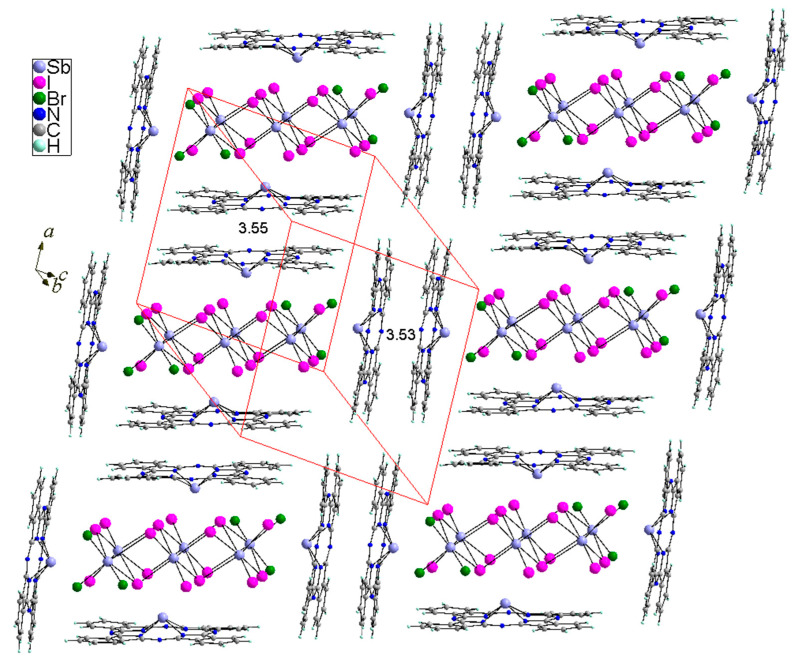
Packing of [(SbPc)_4_(Sb_6_I_16_Br_6_)] showing a stacking structure in **1**.

Each of the two independent (SbPc)^+^ cations interact with the [Sb_6_I_16_Br_6_]^4−^ counterion in a slightly different manner. The Sb4 of (SbPc)+ interacts with three iodine atoms (I1, I2 and I3) while the Sb4B of (SbPc)+ interacts with two iodines (I5 and I7) and one bromine (Br3) with the contacts Sb–I and Sb–Br being much longer than the sum of the covalent radii of Sb (1.39 Å), I (1.40 Å) and Br (1.20 Å) [87] but shorter than the sum of the van der Waals radii Sb and I of ~4.3 Å or Sb and Br of ~4.1 Å [88]. The average Sb–I distance of 3.578 Å and the Sb–Br distance of 3.5941(18) Å between the Sb of (SbPc)^+^ units and I or Br atoms of [Sb_6_I_16_Br_6_]^4−^ complex anion point that the interaction between (SbPc)^+^ units and [Sb_6_I_16_Br_6_]^4−^ anion is intermediate between ionic and covalent. The centrosymmetric [(SbPc)_4_(Sb_6_I_16_Br_6_)] aggregates related by translation in crystal form a stacking structure along the [100] and [111] directions with back-to-back oriented (SbPc)^+^ pairs (Figure 4).

The interplanar N_4-isoindole_–N_4-isoindole_ distance within the stack is ~3.5 Å, which indicates an interaction between the non-planar (SbPc)+ macrocycles with an intermediate force. The π-π interaction and overlapping of the π clouds of (SbPc)^+^ macrocycles in the present crystal **1** is significantly lower compared to that found in the normal planar M(II)Pc structures. Decreasing of π–π interactions together with the ionic character of the (SbPc)^+^ and (Sb_6_I_16_Br_6_)^4−^ units result in improvement to its solubility as well as affects its optical spectroscopic properties.

### 3.3. Hirshfeld Surface Analysis

**Figure 5:**
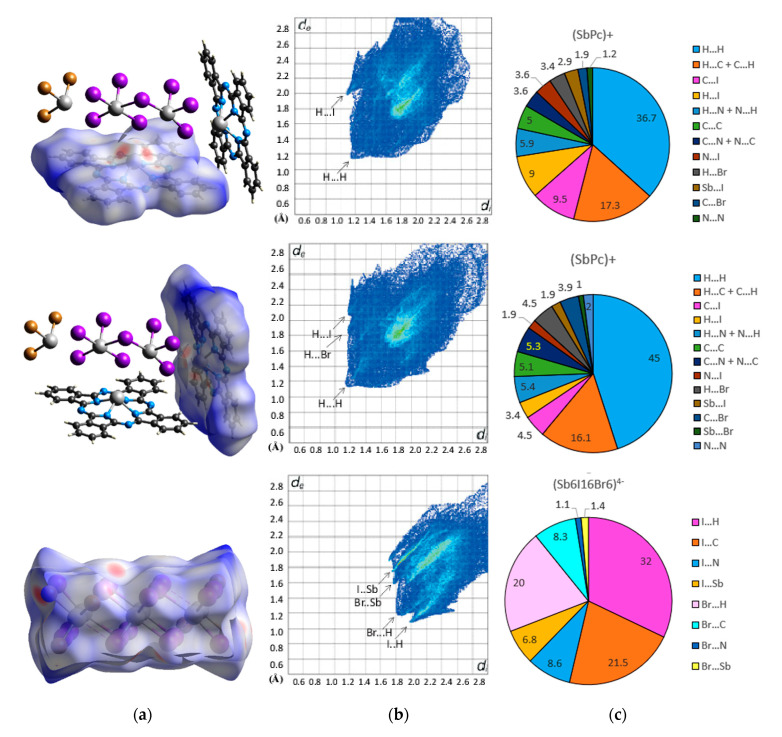
Hirshfeld surface (**a**) and the 2D-fingerprint plots (**b**) for each building units of the crystal **1** and (**c**) percentage contributions of the respective interactions in the HS.

To better understand the nature of the interactions between the components constituting the crystal **1**, the Hirshfeld surface analysis and the analysis of the 2D fingerprint plots were performed. The Hirshfeld surface (HS) analysis and the analysis of 2D fingerprint plots are good tools that not only allow for qualitative analysis of the intermolecular interactions in the crystals [89,90,91,92], but also allow for quantitative analysis, i.e., allow the percentage contribution to the HS surface resulting from particular types of interactions between building blocks of the crystal **1** to be calculated. The 3D Hirshfeld surfaces are mapped via the normalized contact distance (*d_norm_*) relative to both (*d_e_*) and (*d_i_*) and the van der Waals radii of the atoms, where *d_e_* is the distance from a point on the surface to the nearest nucleus outside the surface, and *d_i_* is distance from a point on the surface to the nearest nucleus inside the surface and 2D-fingerprint plots of each units of crystal, i.e., both independent (SbPc)^+^ units and the [Sb_6_I_16_Br_6_]^4−^ counterion, and are shown in Figure 5. Where the atoms make intermolecular contacts closer than the sum of van der Waals radii, the contacts are highlighted in red on the HS mapped with the *d_norm_*; longer contacts are blue and the contacts around the sum of van der Waals radii are white. As can be seen from Figure 5, the HS analysis of both independent (SbPc)^+^ units in the crystal reveals that the (SbPc)^+^ units interact differently. The HS of both (SbPc)^+^ units exhibit that the dominant contacts are H–H interactions (dispersive forces) with a contribution of 36.7% and 45.0% in the HS of (SbPc)^+^ units containing the Sb4 and Sb4B, respectively. The interaction between the (SbPc)^+^ units with the (Sb_6_I_16_Br_6_)^4−^ counterion is manifested in the HS, and for the 2D-fingerprint plots that show the H–I contacts for the (SbPc)^+^ unit containing Sb4, the H–I contact contribution is greater (9.0%) than for the Sb4B unit (3.4%). However, in the HS of the second (SbPc)^+^ unit, the H–Br interactions with a contribution of ~4.5% are observed, but interactions of this type (H–Br) are absent in the HS for the first (SbPc) unit containing Sb4. Similar correlations as for H–I and H–Br are observed in the C–I and C–Br interactions in the HS of the (SbPc)^+^ units. The H–C/C–H and H–N/N–H interactions in the HS with contributions of 17.3% and 5.9%, and 16.1% and 5.4%, respectively, are found in the SbPc units with Sb4 and Sb4B atoms, respectively. The contribution of the π–π interactions between the back-to-back-oriented pair of (SbPc)^+^ units, which correlate well with the C–C and C–N/N–C contacts in the HS of approximately 8.5% and 10.4% for (SbPc)_2_ unit pairs containing Sb4 and Sb4B atoms, respectively, and is 2 ÷ 2.5 times smaller than in normal and planar metallophthalocyanine crystals in which these interactions reach about 22% [93].

The HS of the [Sb_6_I_16_Br_6_]^4−^ unit shows small light-red areas resulting from the interaction of I–Sb and Br–Sb with the surrounding units (SbPc)^+^ with contributions of 6.8% and 1.4% in the HS. However, the main contribution to the HS results from the interactions of I–H and Br–H, which account for more than 50% of the HS area. The contribution in area of the HS of the (Sb_4_I_16_Br_6_)^4−^ anion resulting from the I–C, Br–C, I–N, and Br–N interactions with a total share is about 40%. The deconvolution of the 2D-fingerprint plot illustrating the respective interactions for both independent (SbPc)^+^ units and for (Sb_4_I_16_Br_6_)^4−^ counterion is shown in Figure S2 (in Supplementary Materials).

### 3.4. UV–Vis Spectrosopy

**Figure 6:**
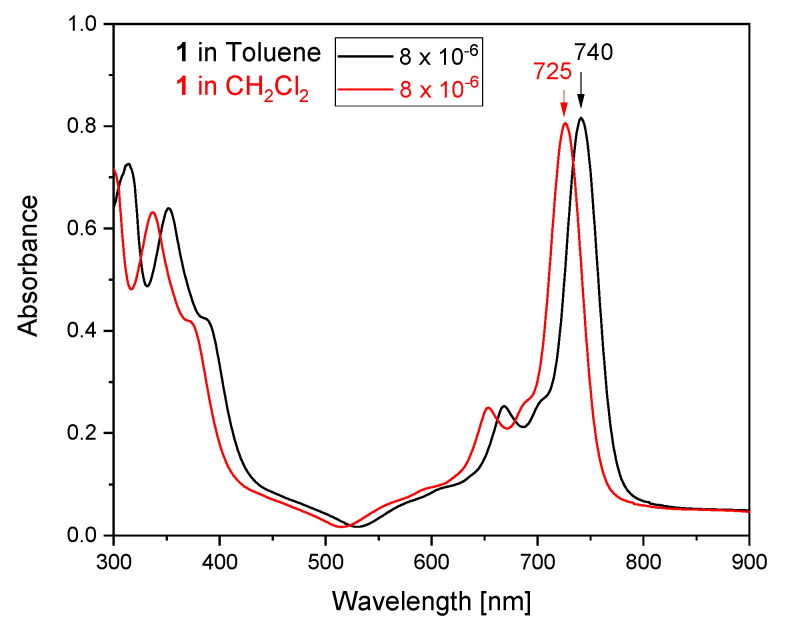
Electronic absorption spectra of antimony(III) phthalocyanine (**1**), [(SbPc)_4_(Sb_6_I_16_Br_6_)], in toluene (black) and CH_2_Cl_2_ (red) solutions.

To characterize the optical properties of the obtained antimony(III) phthalocyanine complex, [(SbPc)_4_(Sb_6_I_16_Br_6_)], the absorption UV–Vis electronic spectra were made in CH_2_Cl_2_ and toluene solutions (Figure 6). The ground state electronic absorption spectra of [(SbPc)_4_(Sb_6_I_16_Br_6_)] in CH_2_Cl_2_ and in toluene solutions show relatively sharp Q bands typical of unaggregated MPc complexes, as has been reported before for other antimony(III)–phthalocyanine derivatives [94,95]. The Q band in the spectrum of the [(SbPc)_4_(Sb_6_I_16_Br_6_)] complex in both solutions is broadened, and the half-width of this band is almost twice as wide as compared to the spectra of normal/flat M(II) Pc complexes [96,97,98], since the Sb(III) is larger than the equilibrium cavity size of the ring and cannot be accommodated without ring expansion; thus, the metal protrudes out of the cavity, forming a non-planar Sb(III) phthalocyanine complex. Thus, the symmetry of the (SbPc)^+^ complex unit in solution becomes *D_4v_* symmetry that is lower than *D_4h_* as for normal/planar MPc complexes in solutions [99]. Lowering of the symmetry of the (SbPc)^+^ allows for some so far forbidden transitions, and consequently, spectral splitting as well as a broadening is observed. The Q-band, an electronic transition from HOMO to LUMO of the phthalocyanine ligand in character, in the spectrum of the SbPc complex **1** in the CH_2_Cl_2_ solution is observed at ~725 nm (logε = 5.33), and the Q band in the spectrum in toluene solution is red shifted to ~740 nm (logε = 5.35), whereas for normal, planar and with D*_4h_* symmetry of metal(II) phthalocyanines, the Q band is observed at almost the same wavelength (~670 nm) [100].

**Figure 7:**
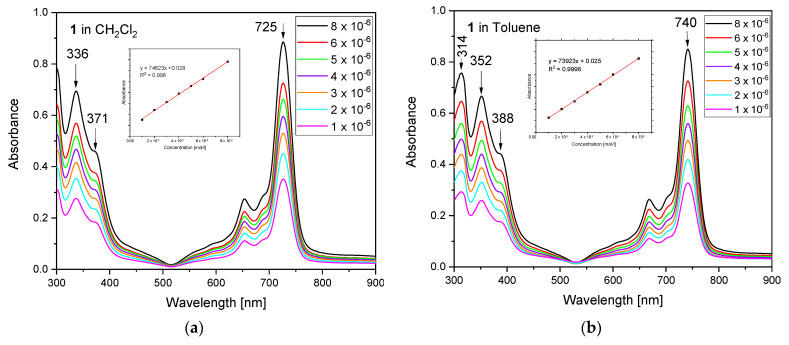
Optical absorption spectra of SbPc-complex **1** in CH_2_Cl_2_ (**a**) and in toluene (**b**) at various concentrations (8 × 10^−6^–1 × 10^−6^ mol/L).

The aggregation behavior of the investigated antimony(III)–phthalocyanine complex (**1**), [(SbPc)_4_(Sb_6_I_16_Br_6_)], was examined by varying the concentrations in both used solvents within the limits of the Beer–Lambert’s law. The aggregation of phthalocyanines, especially the planar metallophthalocyanines occurs in solution through a coplanar association that makes the conversion of a monomer to higher order complexes. However, this antimony(III) phthalocyanine complex, due to the non-planarity of the (SbPc)^+^ unit and the partially ionic character of the complex, is better soluble in the most organic common solvents. The aggregation behavior of the [(SbPc)_4_(Sb_6_I_16_Br_6_)] complex was investigated in CH_2_Cl_2_ and toluene solutions with concentrations between 8 × 10^−6^ mol/L and 10^−6^ mol/L. Both solvents are characterized by a non-zero dipole moment, μ = 1.60 D and μ = 0.36 D for CH_2_Cl_2_ and toluene, respectively [101]. The antimony(III) phthalocyanine complex (**1**) has minimum aggregation in both solvents, as illustrated in Figure 7. With an increase in concentration, the intensity of absorption of the Q-band increased linearly, with no new band and wavelength shifts observed, which clearly evidenced that the aggregate species was not observed.

**Figure 8:**
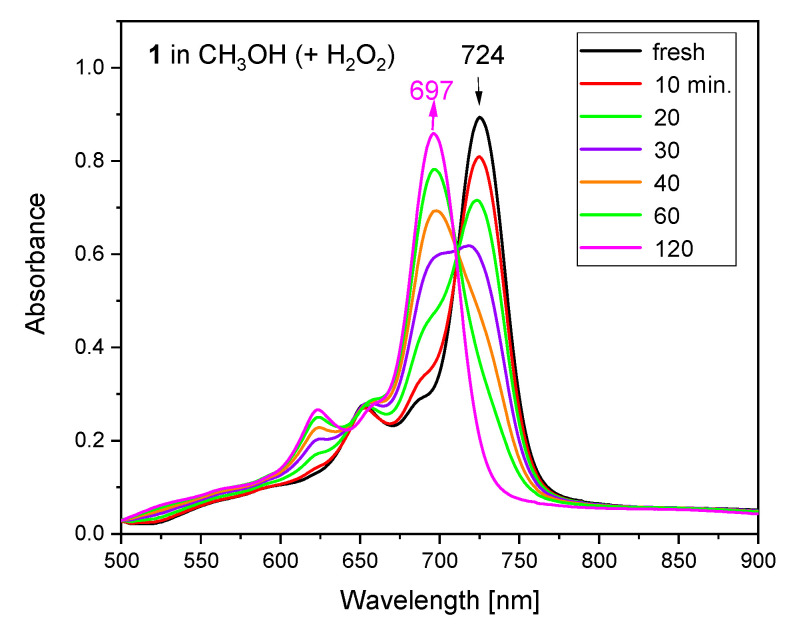
Spectral changes during the oxidation by H_2_O_2_ of 5 × 10^−6^ mol [(SbPc)_4_(Sb_6_I_16_Br_6_)] in methanol. The Q bands at 724 nm and 697 nm are characteristic for the Sb^III^ and for Sb^V^ phthalocyanines, respectively.

The ability of [(SbPc)_4_(Sb_6_I_16_Br_6_)] to oxidize by H_2_O_2_ in a methanol solution was investigated. Hydrogen peroxide as an oxidant of metallophthlocyanines was often used. Therefore, for oxidation of [(SbPc)_4_(Sb_6_I_16_Br_6_)] dissolved in methanol (concentration of 5 × 10^−6^ mol/L) to Sb^V^Pc derivative the H_2_O_2_ was also used. The changes in the absorption spectrum in the range of the Q band (500–900 nm) during the oxidation process were monitored over time (Figure 8).

The Q band characteristic of Sb^III^Pc at 724 nm begins to decrease with the simultaneous appearance and increase in intensity of the Q band characteristic for Sb^V^Pc derivatives. Complete disappearance of the Q band from (Sb^III^Pc)^+^ occurred after approximately 2 h. Oxidation of Sb^3+^ to Sb^5+^ is associated with a reduction of the ionic radius from 0.90 to 0.64 Å [69], with the result that the too large Sb^3+^ ion protruding from the ring plane in (Sb^III^Pc)^+^ after oxidation to Sb^5+^ adapts to the size of the ring cavity. Thus, the Sb^5+^ lies in the plane of the Pc ring. The smaller size and higher electronegativity lead to increasing the conjugation of the Pc’s HOMO with the metal 5 pz orbital [102], which results in lower charge density and energy of the Pc’s HOMO. The effect of this is the observed blue shifting of spectral Q band positions (Figure 8). In the solution after oxidation of (Sb^III^Pc)^+^ with *D_4v_* symmetry by H_2_O_2,_ the [Sb^V^Pc(OH)_2_]^+^ complex with OH groups in the trans positions with approximately *D_4h_* symmetry is formed. A quite similar correlation between the Q bands was observed during the oxidation of the complex (SbPc)(I_3_)∙½I_2_ [68]. This Sb(V)–phthalocyanine complex, [Sb^V^Pc(OH)_2_]^+^, was also suggested by Knӧr [15], who studied the oxidation of (Sb^III^Pc)F by hydrogen peroxide in an ethanol solution.

## 4. Conclusions

A new, low valence antimony–phthalocyanine complex under iodine vapor atmosphere, [(SbPc)_4_(Sb_6_I_16_Br_6_)]—(**1**), was obtained in the crystalline form. In the crystal, the (SbPc)^+^ unit is non-planar, since the Sb(III) is larger than the cavity size of the ring; thus, the metal protrudes out of the cavity, forming a saucer shape. The Hirshfeld surface analysis and the analysis of two-dimensional fingerprint plots show approximately 2.5 times lower π–π interaction between the partially overlapping and saucer-shaped phthalocyanine rings of (SbPc)^+^ units compared to normal, planar MPc’s. This improves its solubility. In the CH_2_Cl_2_ solution, the Q band is observed at 725 nm, whereas in toluene solution this band is red shifted to 740 nm. Since the [(SbPc)_4_(Sb_6_I_16_Br_6_)] as a low valent metal of 15 main group phthalocyanine complex is similar to other Sb^III^Pc derivatives, it does not fluorescence; therefore, it can be used as precursor in obtaining (after oxidation) the high valent Sb^V^Pc complex that is similar to other Sb^V^–phthalocyanine derivatives, showing the fluorescence excitation spectral. Oxidation of the complex (Sb^III^Pc)^+^ with *D_4v_* symmetry yields the [Sb^V^Pc(OH)_2_]^+^ complex with OH groups in the trans positions with approximately *D_4h_* symmetry. It should also be noted that the [(SbPc)_4_(Sb_6_I_16_Br_6_)] before and after the oxidation to the Sb^V^Pc derivative show a characteristic UV–Vis absorption in the near-infrared range of 600–900 nm (i.e., in the therapeutic window), which should be intriguing from the point of view of potential use as an infrared cut-off filter for plasma displays, silicon photodiodes, solar cells, and charge-generating material as well as photosensitizers for semiconductor lasers or for PDT.

## Figures and Tables

**Table 1:** Crystal data and final refinement parameters for **1**.

	[(Sb^III^Pc)_2_(Sb_2_I_8_)(SbBr_3_)]—(1)
Formula	C_64_H_32_Br_3_I_8_N_16_Sb_5_
Molecular weight	2888.73
Temperature (K)	295(2)
Crystal system	Triclinic
Space group	$P\bar{1}$
*a*, *b*, *c* (Å)	14.399(2), 15.344(3), 18.992(3)
*α*, *β*, *γ* (°)	95.64(2), 96.89(2), 113.93(2)
*V* (Å^3^)	3757.4(12)
Z	2
*D_cal_ (*gcm^−3^*)*	2.553
Crystal size (mm^3^)	0.250 × 0.125 × 0.080
μ (mm^−1^)	6.707
Absorption correction	multi-scan
T_max._/T_min._	0.4754/1.000
Total/Unique/Obs refls	23,647/14,738/7721
*R*_int_	0.0271
*R*, [*F*^2^ > 2σ(*F*^2^)] ^1^	0.0476
*wR* [*F*^2^ all refls] ^2^	0.0957
*S*	1.001
Δρ_max_, Δρ_min_ (e·Å^−3^)	2.072, −1.286

^1^ *R* = Σ ||*F*_o_| − |*F*_c_||/Σ*F*_o_; ^2^ *wR* = {Σ [*w*(*F*_o_^2^ − *F*_c_^2^)^2^]/Σ*wF*_o_^4^}^½^; *w*^−1^ = σ^2^(*F*_o_^2^) + (*aP*)^2^ where *P* = (*F*_o_^2^ + 2*F*_c_^2^)/3. The *a* parameter is 0.0263.

**Table 2:** Selected bond lengths and angles (Å, °) in crystal of **1**.

(SbPc)^+^ Cations					
Sb4–N2		2.191(8)	Sb4B–N2B		2.202(7)
Sb4–N4		2.178(8)	Sb4B–N4B		2.181(7)
Sb4–N6		2.190(9)	Sb4B–N6B		2.206(8)
Sb4–N8		2.206(8)	Sb4B–N8B		2.146(8)
N2–Sb4–N4		78.0(3)	N2B–Sb4B–N4B		77.6(3)
N4–Sb4–N6		79.4(3)	N4B–Sb4B–N6B		77.7(3)
N6–Sb4–N8		77.5(3)	N6B–Sb4B–N8B		78.5(3)
N2–Sb4–N8		77.7(3)	N2B–Sb4B–N8B		78.4(3)
Deviation of Sb from N_4_-plane		0.992(1)	Deviation of Sb from N_4_-plane		0.994(1)
**(Sb_6_I_16_Br_6_)^4−^ Anion**					
**Terminal Sb–I**					
Sb1–I7	2.7668(13)	Sb1–I8	2.6965(12)	Sb2–I6	2.7374(11)
**Bridged μ_2_**					
Sb1–I3	3.2329(11)	Sb2–I3	2.9833(13)	Sb1–I5	2.8744(11)
Sb3–I5 *^i^*	3.4481(10)	Sb2–I4	2.8302(10)	Sb3–I4	3.31901(10)
**Bridged μ_3_**					
Sb1–I1	3.1765(14)	Sb2–I1	3.1856(11)	Sb2*^i^*–I1	3.3610(10)
Sb2–I2	3.0061(13)	Sb1*^i^*–I2	3.4281(12)	Sb3–I2	3.3531(11)
**Terminal Sb–Br**					
Sb3–Br1	2.6888(12)	Sb3–Br2	2.5659(14)	Sb3–Br3	2.6321(15)
I8–Sb1–I7	96.78(4)	I8–Sb1–I5	90.48(4)	I7–Sb1–I5	98.42(4)
I8–Sb1–I1	93.88(4)	I7–Sb1–I1	166.21(3)	I5–Sb1–I1	90.20(3)
I8–Sb1–I3	91.83(3)	I7–Sb1–I3	85.88(4)	I5–Sb1–I3	174.85(3)
I1–Sb1–I3	85.05(3)	I6–Sb2–I4	97.06(3)	I6–Sb2–I3	90.66(4)
I4–Sb2–I3	88.68(3)	I6–Sb2–I2	91.49(3)	I4–Sb2–I2	93.83(4)
I3–Sb2–I2	176.48(3)	I6–Sb2–I1	90.88(3)	I4–Sb2–I1	171.80(3)
I3–Sb2–I1	89.16(3)	I2–Sb2–I1	88.02(3)	Br2–Sb3–Br3	92.11(5)
Br2–Sb3–Br1	97.60(5)	Br3–Sb3–Br1	96.8 (5)	Sb1–I1–Sb2	91.46(3)
Sb2–I3–Sb1	94.17(3)				
**Short Contacts between (SbPc)^+^ Units and (Sb_6_I_16_Br_6_)^4−^ Anion**					
Sb4–I1 *^i^*	3.5083(13)	Sb4–I2*^i^*	3.6471(13)	Sb4–I3	3.6115(14)
Sb4B–I5	3.6283(14)	Sb4B–I7	3.4969(11)	Sb4B–Br3*^i^*	3.5941(18)

Symmetry Code: (*i*) −x, −y, −z + 1.

## Supplementary Materials

The following supporting information can be downloaded at: https://www.mdpi.com/article/10.3390/molecules27061839/s1. Additional material contains the experimental IR spectrum of **1**, full optimized parameters for (SbPc)^+^ unit and figures illustrating the deconvolution of the 2D-figerprint plots for individual types of interactions in **1**. CCDC No. 2131217 contains the supplementary crystallographic data for **1**. These data can be obtained free of charge via http://www.ccdc.cam.ac.uk/conts/retrieving.html (accessed on 2 January 2022) or from the Cambridge Crystallographic Data Centre, 12 Union Road, Cambridge CB2 1EZ, UK; Fax: +44-1223-336-033; or email: deposit@ccdc.cam.ac.uk.
