# Supplementary material for: Synthesis, Structure, and UV–Vis Characterization of Antimony(III) Phthalocyanine: [(SbPc)2(Sb2I8)(SbBr3)]2
Source: Molecules. 2022 Mar 11;27(6):1839. doi: 10.3390/molecules27061839 (PMC8950410; doi:10.3390/molecules27061839)
Supplement: Supplementary file 1 [file molecules-27-01839-s001.zip › molecules-1586248-supplementary.pdf]

Synthesis, structure, and UV-Vis characterization of antimony(III) phthalocyanine: $[(SbPc)_2(Sb_2I_8)(SbBr_3)]_2$

Ryszard Kubiak and Jan Janczak*

Institute of Low Temperature and Structure Research, Polish Academy of Sciences, Okólna 2, 50-422 Wrocław, Poland

* Correspondence: j.janczak@intibs.pl

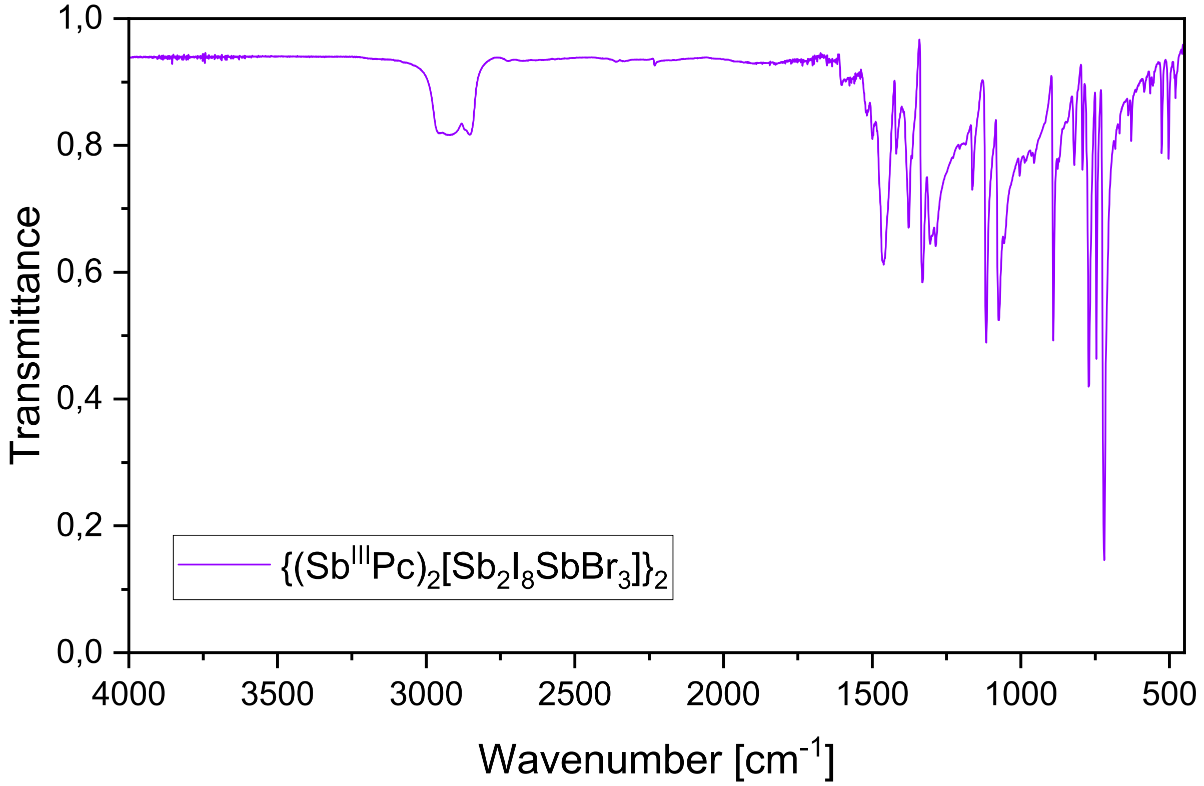

Figure S1. IR spectrum of $\{(Sb^{III}Pc)_2(Sb_2I_8SbBr_3)\}_2$ - (1) (nujol mull).

Table S1. Optimized parameters for $(SbPc)^+$ unit (Å, °).

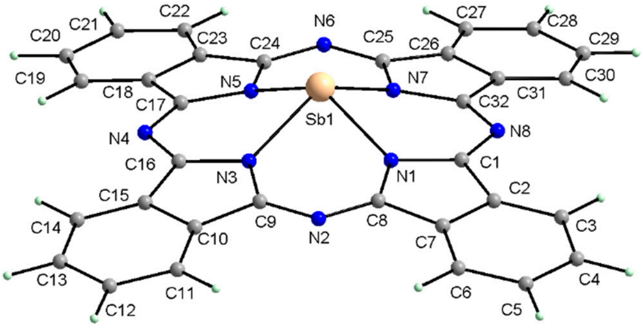

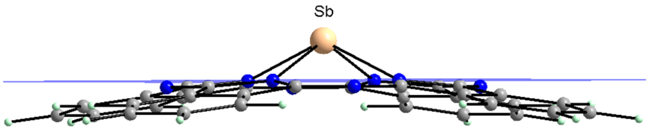

N_4-isoindole (N1,N3,N5,N7) plane is marked in blue.
The displacement of Sb from N_4-plane is 0.886 Å.

Sb1–N1	2.206	Sb1–N3	2.206	Sb1–N5	2.206	Sb1–N7	2.206
N1–C1	1.442	C1–C2	1.477	C2–C3	1.419	C3–C4	1.414
C4–C5	1.428	C5–C6	1.414	C6–C7	1.419	C7–C2	1.434
C7–C8	1.477	C8–N1	1.442	C8–N2	1.383	N2–C9	1.383
C9–N3	1.442	C9–C10	1.477	C10–C11	1.419	C11–C12	1.414
C12–C13	1.428	C13–C14	1.414	C14–C15	1.419	C15–C10	1.434
C15–C16	1.477	C16–N3	1.442	C16–N4	1.383	N4–C17	1.383
C17–N5	1.442	C17–C18	1.477	C18–C19	1.414	C19–C20	1.419
C20–C21	1.428	C21–C22	1.414	C22–C23	1.419	C23–C18	1.434
C23–C24	1.477	C24–N5	1.442	C24–N6	1.383	N6–C25	1.383
C25–N7	1.442	C25–C26	1.477	C26–C27	1.419	C27–C28	1.414
C28–C29	1.428	C29–C30	1.414	C30–C31	1.419	C31–C26	1.434
C31–C32	1.477	C32–N7	1.442	C32–N8	1.383	N8–C1	1.383
C–H 1.106							
N1–Sb1–N3	80.71	N3–Sb1–N5	80.71	N5–Sb1–N7	80.71	N7–Sb1–N1	80.71
C1–N1–C8	108.24	C8–N2–C9	119.06	C9–N3–C16	108.24	C16–N4–C17	119.07
C17–N5–C18	108.24	C24–N6–C25	119.05	C25–N7–C26	108.24	C32–N8–C1	119.06
N1–C1–C2	108.09	C1–C2–C3	131.15	C2–C3–C4	117.64	C3–C4–C5	121.31
C4–C5–C6	121.31	C5–C6–C7	117.64	C6–C7–C8	131.15	C7–C8–N2	122.10
N2–C9–C10	122.09	C9–C10–C11	131.15	C10–C11–C12	117.64	C11–C12–C13	121.31
C12–C13–C14	121.32	C13–C14–C15	117.64	C14–C15–C16	131.17	C15–C16–N4	122.10
N4–C17–C18	122.09	C17–C18–C19	131.15	C18–C19–C20	117.63	C19–C20–C21	121.31
C20–C21–C22	121.31	C21–C22–C23	117.64	C22–C23–C24	131.17	C23–C24–N6	122.10
N6–C25–C26	122.09	C25–C26–C27	131.15	C26–C27–C28	117.64	C27–C28–C29	121.31
C28–C29–C30	121.31	C29–C30–C31	117.64	C30–C31–C32	131.17	C31–C32–N8	122.10
Sb1–N1–C1–C2	161.55	Sb1–N3–C9–C10	161.60	Sb1–N5–C17–C18	161.55		
Sb1–N7–C25–C26	161.61	N1–C1–C2–C3	179.02	C1–C2–C3–C4	-179.17		
C2–C3–C4–C5	0.68	C3–C4–C5–C6	0.00	C4–C5–C6–C7	-0.68		
C5–C6–C7–C8	179.16	C6–C7–C8–N2	-1.50	C7–C8–N2–C9	-170.77		
N2–C9–C19–C11	1.46	C9–C10–C11–C1	-179.16	C10–C11–C12–C13	0.68		
C11–C12–C13–C14	0.00	C12–C13–C14–C15	-0.68	C13–C14–C15–C16	179.16		
C14–C15–C16–N4	-1.48	C15–C16–N4–C17	-170.76	C16–N4–C17–C18	170.77		
N4–C17–C18–C19	1.48	C17–C18–C19–C20	-179.14	C18–C19–C20–C21	0.68		
C19–C20–C21–C22	0.01	C20–C21–C22–C23	-0.68	C21–C22–C23–C24	179.16		
C22–C23–C24–N6	-1.52	C23–C24–N6–C25	-170.76	C24–N6–C25–C26	170.75		
N6–C25–C26–C27	1.46	C25–C26–C27–C28	-179.18	C26–C27–C28–C29	0.68		
C27–C28–C29–C30	0.00	C28–C29–C30–C31	-0.68	C29–C30–C31–C32	179.16		
C30–C31–C32–N7	-179.04	C30–C31–C32–N8	-1.48	C31–C32–N8–C1	-170.75		
C32–N8–C1–N1	-6.19						

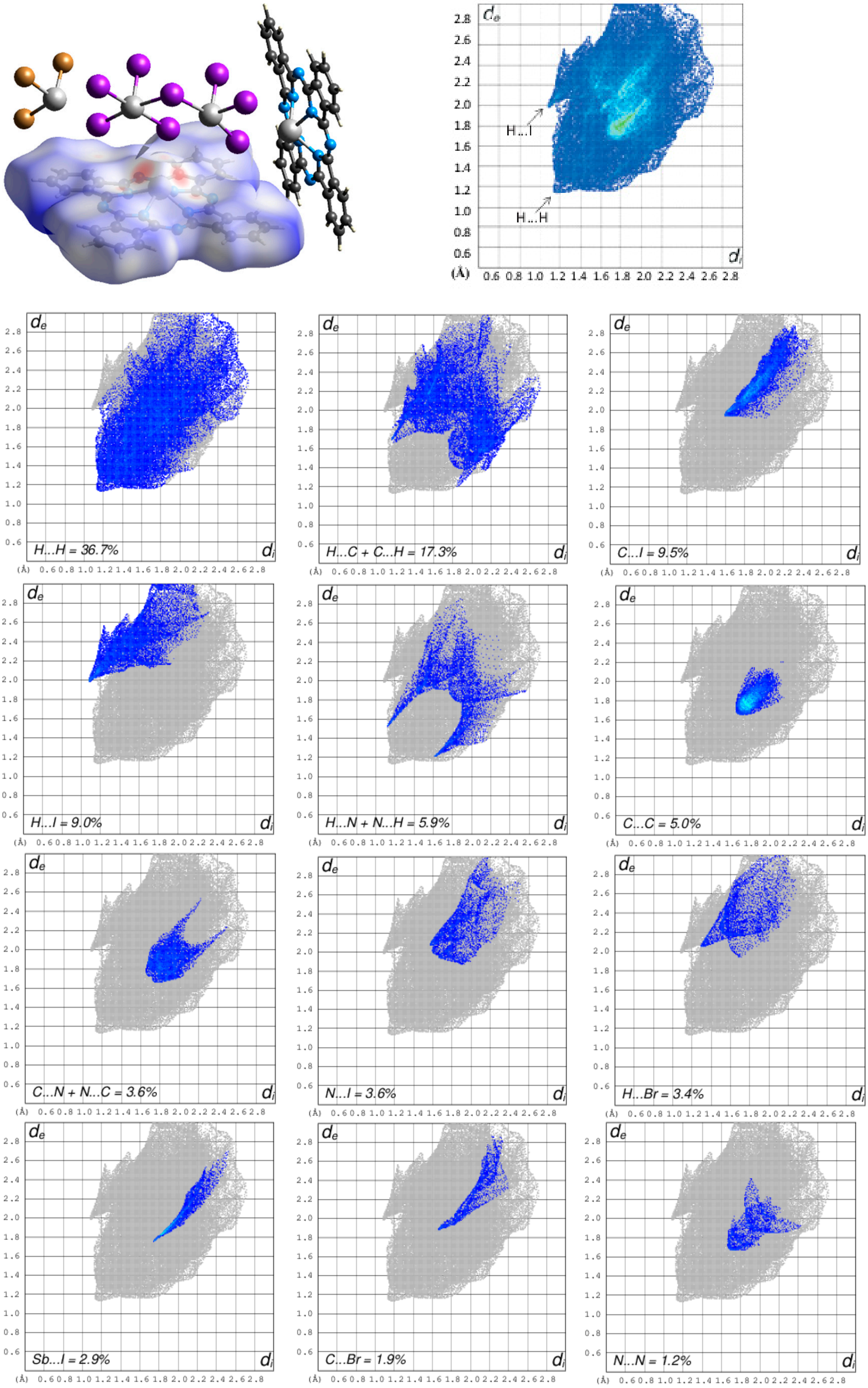
H...I
H...H
H...H = 36.7%
H...C + C...H = 17.3%
C...I = 9.5%
H...I = 9.0%
H...N + N...H = 5.9%
C...C = 5.0%
C...N + N...C = 3.6%
N...I = 3.6%
H...Br = 3.4%
Sb...I = 2.9%
C...Br = 1.9%
N...N = 1.2%

(**a**)

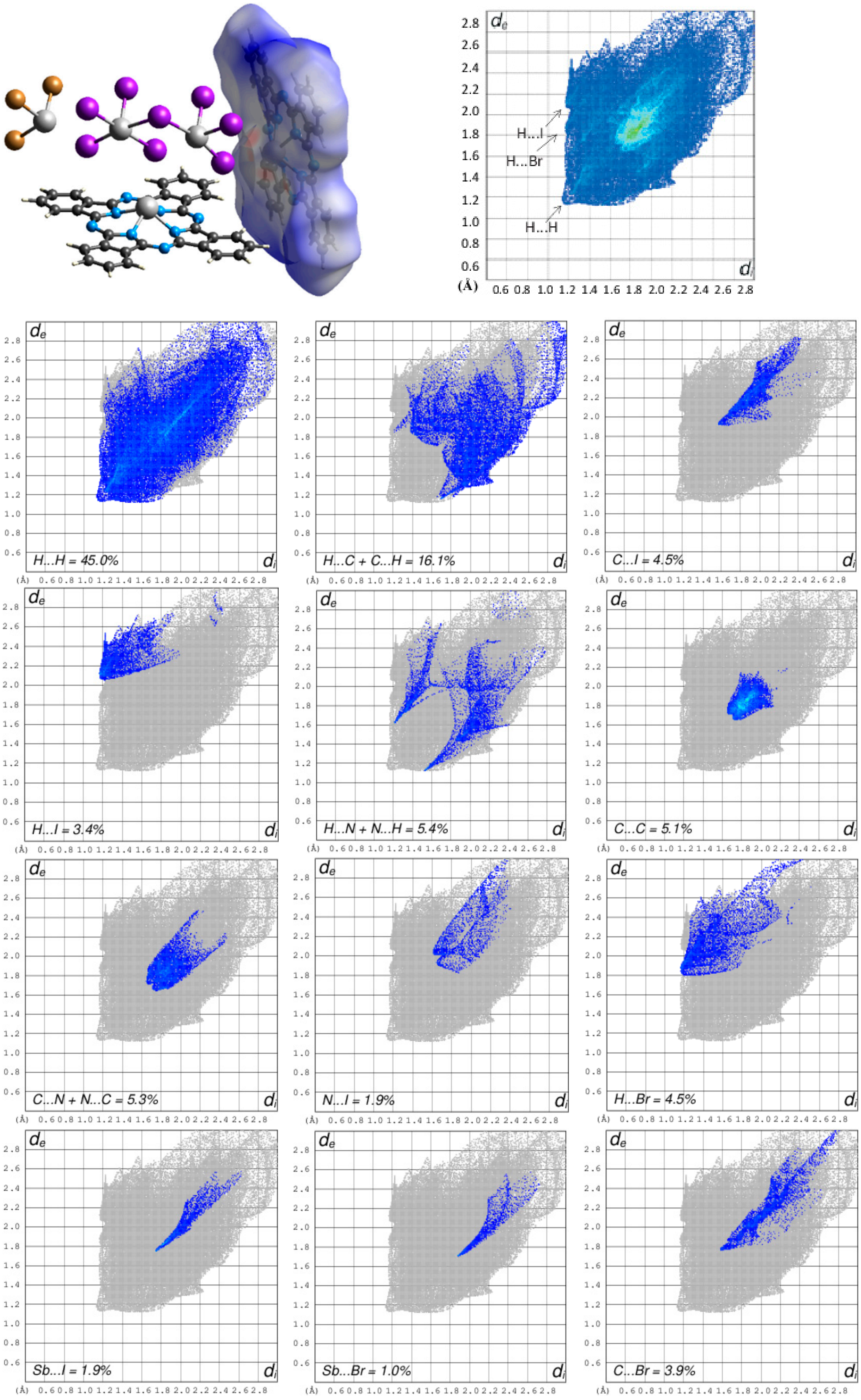
H...I
H...Br
H...H
H...H = 45.0%
H...C + C...H = 16.1%
C...I = 4.5%
H...I = 3.4%
H...N + N...H = 5.4%
C...C = 5.1%
C...N + N...C = 5.3%
N...I = 1.9%
H...Br = 4.5%
Sb...I = 1.9%
Sb...Br = 1.0%
C...Br = 3.9%

(**b**)

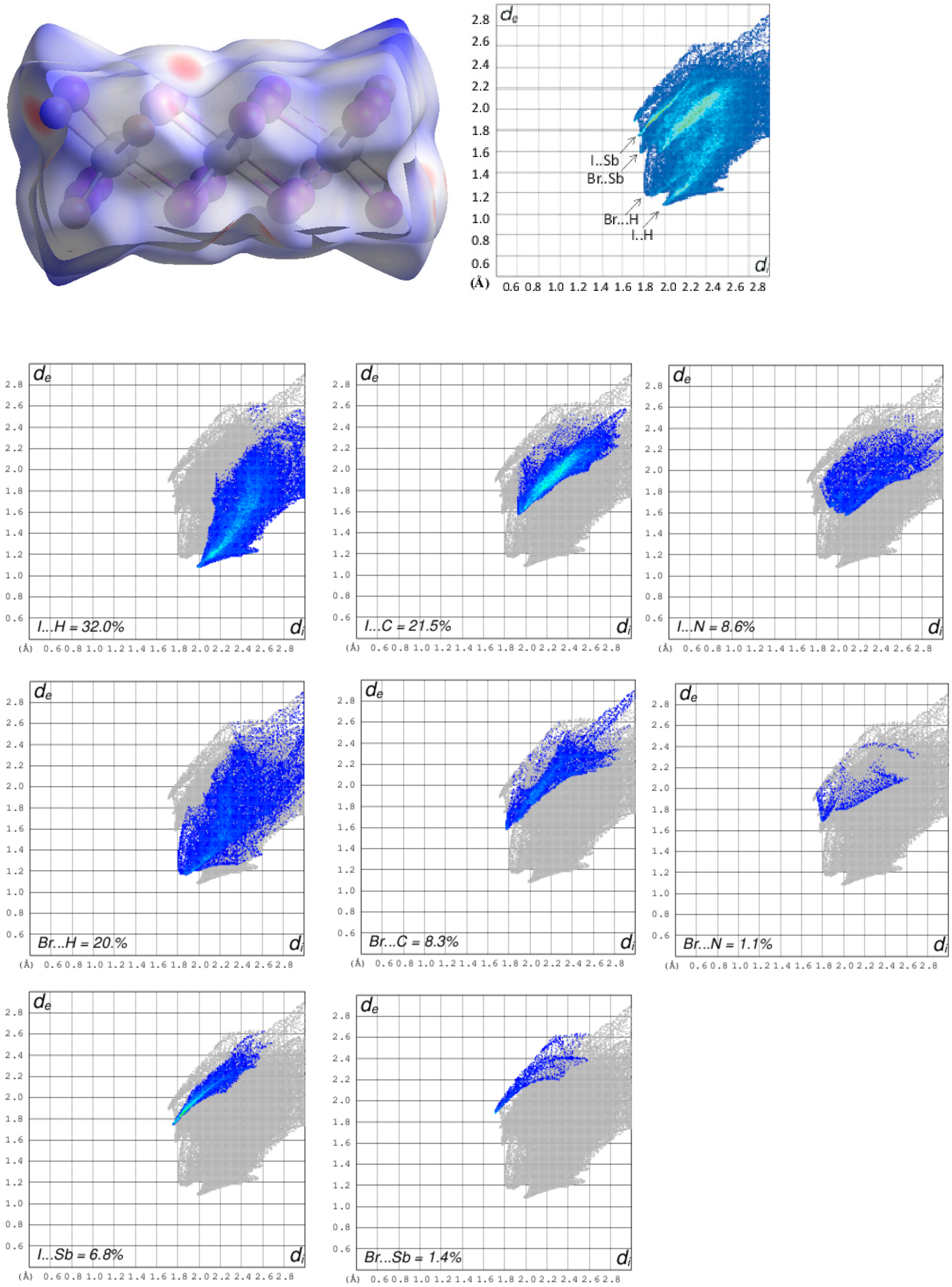

(c)

Figure S2. (**a**) The deconvolution of the 2D fingerprint plot illustrating the percentage of the respective interactions in the HS for (SbPc)$^+$ unit with Sb4 in crystal **1**. (**b**) The deconvolution of the 2D fingerprint plot illustrating the percentage of the respective interactions in the HS for (SbPc)$^+$ unit with Sb4B in crystal **1**. (**c**) The deconvolution of the 2D fingerprint plot illustrating the percentage of the respective interactions in the HS for $(Sb_6I_{16}Br_6)^{4-}$ anionic unit in crystal **1**.
